# Feasibility of ^18^FDG PET in the cardiac inflammation

**DOI:** 10.1007/s10554-020-02056-4

**Published:** 2020-10-15

**Authors:** Sebastian Osiecki, Maciej Sterliński, Marta Marciniak-Emmons, Mirosław Dziuk

**Affiliations:** 1 Department of Nuclear Medicine, Military Institute of Medicine, 128 Szaserów St, 04-141 Warsaw, Poland; 2 Affidea Mazovian PET/CT Medical Centre, 128 Szaserów St, 04-349 Warsaw, Poland; 3 Cardinal Wyszynski National Institute of Cardiology, Warsaw, Poland

**Keywords:** Endocarditis, Sarcoidosis, Positron emission tomography, Fluorodeoxyglucose, Nuclear imaging

## Abstract

The aim of the study was to assess the feasibility of ^18^FDG PET in cardiac inflammation with a particular focus on the delayed scan. Thirty-five consecutive ^18^FDG PET scans of patients with suspected or confirmed cardiac inflammation were retrospectively reviewed. The patients were referred for PET because of endocarditis (n = 16) or sarcoidosis (n = 19). Among them four patients had two consecutive for follow up and treatment control (two patients with sarcoidosis, two with endocarditis). In all of the cases a standard head to mid-thigh scan was performed 45–60 min after ^18^FDG injection as well as a delayed heart scan 1 h after the standard imaging was performed. ^18^FDG PET confirmed active inflammation in 10 out of 35 scans. Delayed scans in positive cases showed SUVmax value increase, but did not have an impact on the result, neither they did in negative cases—no significant differences between standard and delayed scan were found. Interestingly in 5 out of 14 cases with suspected endocarditis PET revealed the extracardiac inflammation focus, thus changing initial diagnosis. ^18^FDG PET also indicated which prosthesis caused inflammation if there were many. In the sarcoidosis group the aim was to confirm or exclude heart involvement (13 scans) or to assess the response to the steroid therapy (6 scans) in patients with previously confirmed sarcoidosis. PET revealed active heart disease in 3 initial scans, and 1 follow up scan. ^18^FDG PET is a valuable imaging method for the cardiac inflammation assessment. It adequately localises the active inflammation site. Also, since it is a whole-body scan it may detect the extracardiac inflammation foci, which in some cases may change the initial diagnosis. In our study the delayed scans showed no added value.

## Introduction

Nowadays the ^18^FDG PET (fluorodeoxyglucose positron emission tomography) imaging is most often used for oncology imaging. However, it is also a valuable tool for inflammation evaluation. This work focuses on cardiac inflammation: infective endocarditis or sarcoidosis.

Sarcoidosis is a rare granulomatous disease which affects mainly the lungs and the mediastinal lymph nodes. Heart involvement is seen in 5 to 25% of cases, less frequently in the Caucasians and more frequently in the Asians [1, 2] Cardiac sarcoidosis symptoms depend on the disease extent and may vary from asymptomatic to severe arrhythmias and heart failure. The gold standard for the diagnosis is a biopsy and a histopathological evaluation. However, it often gives false-negative results due to the focal nature of the disease and a possible sampling error [3]. Imaging techniques used for cardiac sarcoidosis diagnosis are electrocardiography, echocardiography, MRI (magnetic resonance imaging) and PET (positron emission tomography) [2].

Infective endocarditis is a potentially fatal disease. Patients at the highest risk are ones after an implantation of prosthetic materials and with heart diseases. [4] Diagnosis is often difficult to pose as patients often present other co-morbidities or other possible causes of infection. If a prosthesis is the source of endocarditis treatment may require surgical removal. Knowing the possible complications the accurate diagnosis of inflammation source is crucial. Primary imaging technique for endocarditis is the echocardiography. Whenever confirmation is required or the cardiac echo result is equivocal other modalities including nuclear imaging are of assistance. In the literature the ^18^FDG PET showed a 100% negative predictive value. [5].

The mechanism behind PET in cardiac inflammatory diseases is fluorodeoxyglucose uptake in white blood cells, which accumulate in the site of infection [4]. Yet, as the physiological uptake in the myocardium may cloud the inflammation focus, the myocardium needs to be suppressed by means of an appropriate patient preparation. It requires an adequate imaging protocol as well [2].

The procedure protocols vary between centres. For endocarditis ESC guidelines from2015 states that PET/CT is generally performed using a single acquisition time point (generally at 1 h) after administration of ^18^F-FDG [4].In case of sarcoidosis many centres rely on Japanese Society of Nuclear Cardiology recommendations, which state that 60–90 min interval (ideally 90 min) is optimal [2]. The aim of the study was to assess the feasibility of the ^18^FDG PET imaging in the cardiac inflammation with a particular focus on differences resulting from a different time interval between ^18^FDG injection and image acquisition.

## Materials and methods

Thirty-five consecutive ^18^FDG PET scans of patients with suspected or confirmed cardiac inflammation were retrospectively reviewed. Scans were performed between January 2019 and May 2020. Patients age ranged from 14 to 79, there were 6 females and 24 males enrolled. Four patients had two studies performed as a follow-up/treatment response control. The patients were referred for PET because of endocarditis (n = 16) or sarcoidosis (n = 19). Patient preparation included fasting for 6 h or more, with the exception of water intake. Standard head to mid-thigh scans were performed 45–90 min after injection. An hour after the standard scan the delayed heart scan was performed. Radiotracer activities were adjusted to body mass and ranged from 201 to 451 MBq. Acquisitions were performed on Discovery 710 hybrid PET/CT scanner (GE Healthcare, US) with low dose unenhanced CT for anatomical imaging and attenuation correction. Images were reviewed on a dedicated AW software (GE Healthcare, US) SUVmax (maximum standardised uptake value) values normalised to the lean body mass were measured. If foci of increase uptake were present each was measured, in negative scans highest SUVmax value over heart would be taken into consideration. Statistical comparison was performed with Wilcoxon test. In preparation for the scan patients were asked to fast overnight. All scans were reviewed by at least two experienced nuclear medicine specialists.

## Results

### Endocarditis group

Fourteen patients with suspected infectious endocarditis were enrolled, two had also a follow-up PET scans to assess response to the antibacterial treatment, which gives a total number of 16 scans reviewed.

Among 14 primary scans endocarditis was confirmed in four cases with uptake foci in:

- mitral valve prosthesis,
- aortic valve graft (two cases),
- ventricular patch (patient after complicated heart defect correction).

**Fig. 1:**
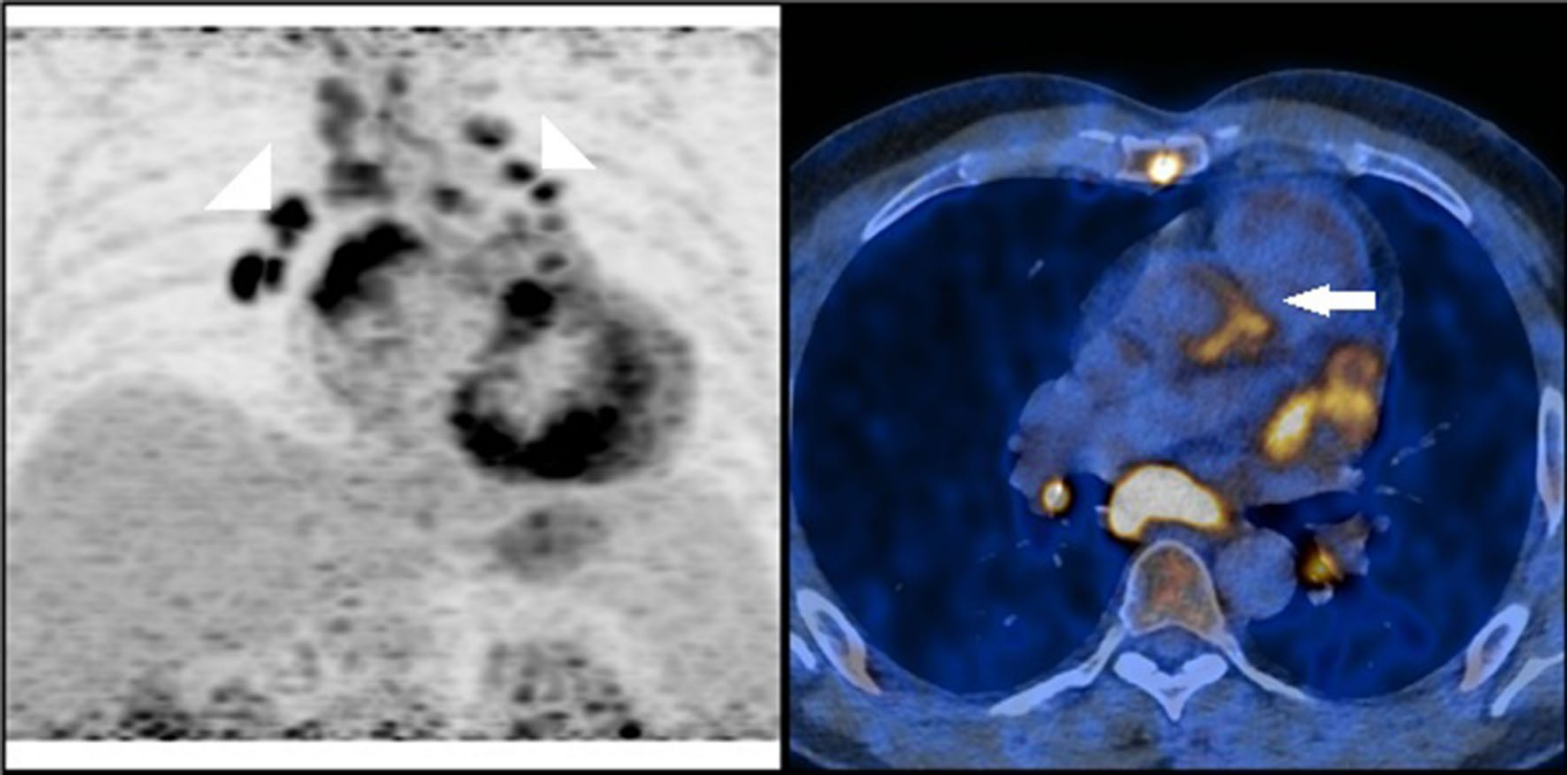
A 36-year-old patient with endocarditis, after mitral and aortal valve replacement. PET scan shows uptake in the aortic graft and in the mediastinal lymph nodes, no uptake in mitral valve is seen

**Fig. 2:**
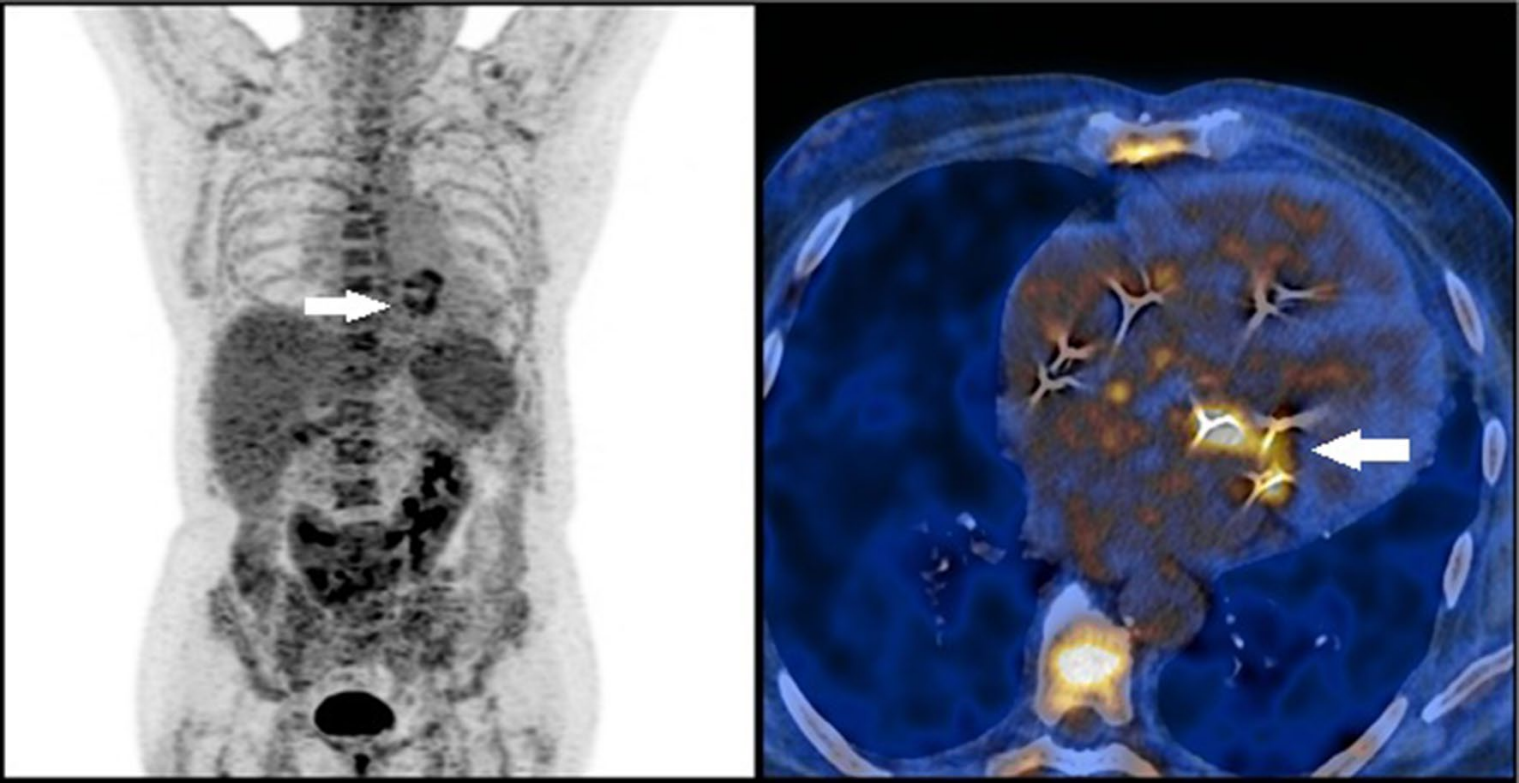
A 79-year-old patient with an endocarditis, after the CRT implantation and the mitral valve replacement. PET scan revealed mitral valve to be the source of inflammation

In two of these cases patients had more than one prosthesis (Figs. 1 and 2). Patient in Fig. 1 had undergone both mitral and aortic valve replacement. Patient in Fig. 2 had undergone a mitral valve replacement and a cardiac resynchronisation therapy (CRT) implantation. ^18^FDG PET clearly indicated which of the two prostheses was infected and, potentially, which ought to be removed.

Follow-up confirmed our diagnoses, with two patients eventually qualified for surgery and two treated pharmacologically with good response to intensive antibiotic therapy.

**Fig. 3:**
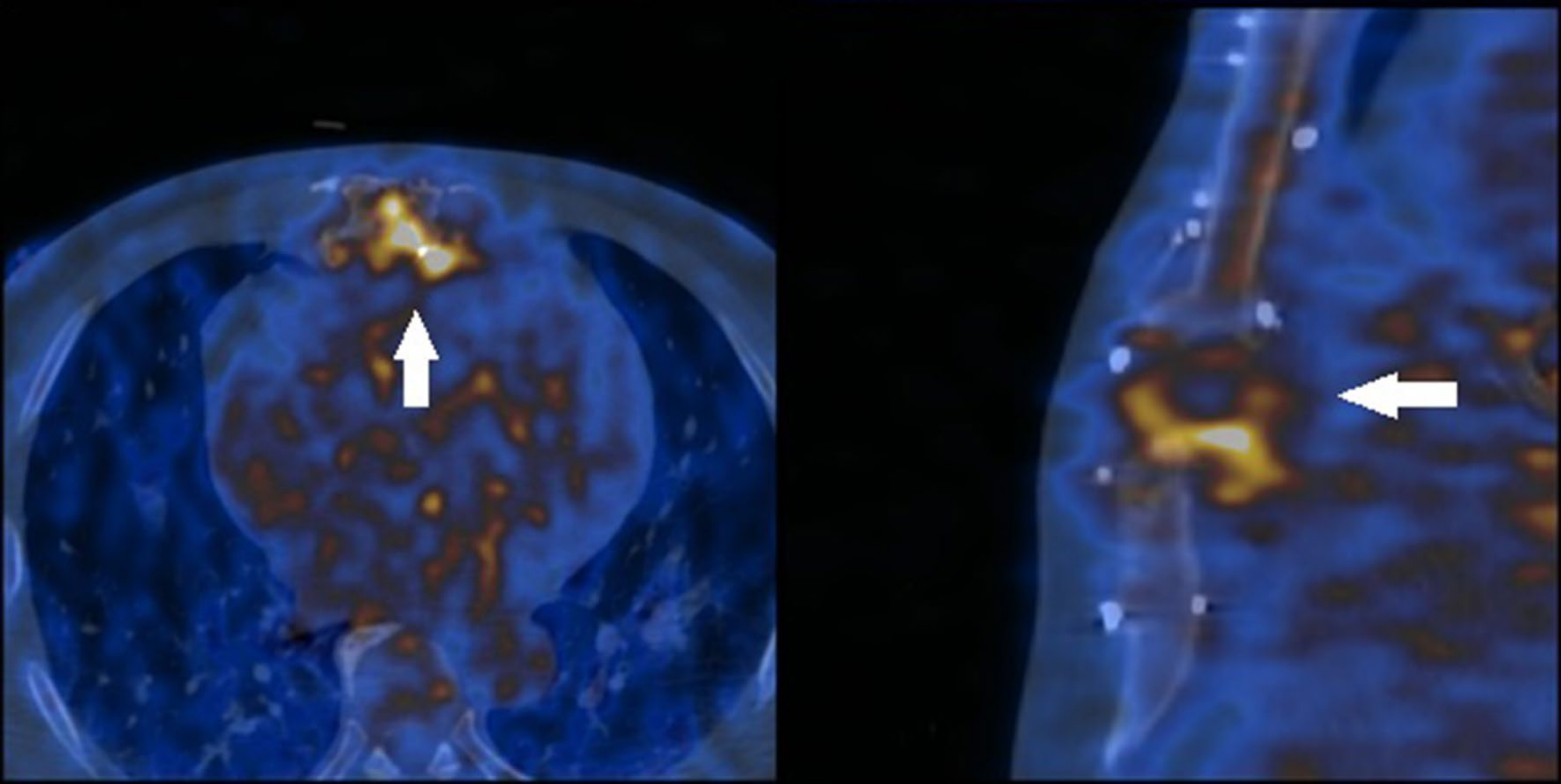
A 57- year-old patient after the aortic valve replacement. PET scan revealed possible infective sternal dehiscence

In 5 of the cases with suspected endocarditis PET scan changed the initial diagnosis excluding endocarditis as a cause of infection. Inflammation foci were found:

- in the lungs (two cases)—treated adequately, one of these patients had also chemotherapy port removed (negative for inflammation in PET) with negative microbiological culture test afterwards,
- in the lymph nodes and the spleen (susp. Lymphoproliferation), in follow-up Stills disease was diagnosed with good response to steroids and methotrexate,
- around the stitches in the aorta (patient after aortic valve replacement)—this patient was lost to follow-up,
- in the sternum (susp. infectious sternal dehiscence)—in follow-up patient responded well to intensive antibiotic therapy (Fig. 3).

Two follow-up PET scans of patients with endocarditis from Figs. 1 and 2 showed no significant improvement after the antibiotic treatment. Yet, the patient in Fig. 2 showed lesser uptake in the mediastinal lymph nodes in the second scan.

Remaining 5 cases consisted mostly of the patients with a suspected mitral valve infection and one case of a suspected pacemaker electrode infection. ^18^FDG PET scans were negative—no foci indicative of inflammation have been found. In follow-up two of the patients had implant replaced (one mitral valve and one pacemaker). Mitral valve was negative for microbiological culture and result of pacemaker test was lost to follow-up. ^18^FDG PET results are summarised in Table 1.

**Table 1:** PET results in patients with suspected infectious endocarditis

	Age	Gender	Problem	Heart in standard scan, additional findings	Heart in delayed scan	Conclusion
1	69	f	Susp. mitral valve endocarditis	Calcifications in mitral valve, SUVmax = 6,9	SUVmax = 4	Overcorrection, no inflamation
2	39	m	After aortic valve replacement	Aortic valve uptake, SUVmax = 5,3	SUVmax = 6	Endocarditis confirmed
3	44	f	Susp. Infection on chemotherapy catheter	Moderately increased uptake around catheter SUVmax = 2,3, lung lesions	Same as standard	Lung inflamation
4	14	m	After correction of complicated heart defect	Focal uptake in tunelising patch in left ventricle, SUVmax = 2,5	SUVmax = 2,7	Endocarditis confirmed
5	19	m	FUO	No active cardiac lesions; active lymph nodes and spleen	Same as standard	Susp. lymhoproliferative process
6	66	m	After removal of ICD due to endocarditis	Physiological uptake in heart, active lesions in lungs	Same as standard	Inflamatory/sarcoid lung disease
7	47	m	After aortic valve replacement	No active cardiac lesions, active lesions around stitches in aorta, SUVmax = 4,1	SUVmax = 5,4	Inflamation in aorta - around stitches
8	58	f	Endocarditis suspected in echocardiography	No active lesions	Same as standard	Endocarditis not confirmed
9	57	m	After aortic valve replacement	Moderatly increased uptake on aortic valve, SUVmax = 3,6, active lesions in sternum	SUVmax = 3,5	Susp. Infective sternal dehiscence, valve image equivocal
10	54	f	After mitral valve replacement	No active lesions	Same as standard	Endocarditis not confirmed
11	72	m	Suspected mitral valve endocarditis	High myocardial uplake close to mitral valve, SUVmax = 5,7	SUVmax = 6	Not univocal for endocarditis
12	18	m	Susp. Endocarditis from pacemaker electrode	Uptake in epicardial electrode SUVmax = 2,3, no uptake on endocavital electrode	SUVmax = 2,8	Endocarditis not confirmed
13	36	m	After aortic and mitral valve replacement	High uptake in aortic valve SUVmax = 6,5, uptake in mitral valve not increased	SUVmax = 10,7	Endocarditis on aortic valve, mitral valve clear
13	Second PET		Follow-up scan after antibiotic treatment	High uptake in aortic valve SUVmax = 6,3, uptake in mitral valve not increased	SUVmax = 7,2	Bad response to treatment
14	79	m	After pacemaker implantation and mitral valve replacement	High uptake in mitral valve, SUVmax = 4,7; pacemaker – reactive uptake	SUVmax = 5	Mitral valve inflamation, pacemaker clear
14	Second PET		Follow-up scan after antibiotic treatment	Mitral valve uptake SUVmax = 4,5	SUVmax = 4,9	Bad response to treatment

### Sarcoidosis group

Since 2019 to May 2020 sixteen patients had a total of 19 ^18^FDG PET scans due to sarcoidosis with a suspected heart involvement. Most of the patients referred for PET scan had already had a confirmed sarcoidosis. The aim of the PET to confirm or exclude heart involvement (13 scans) or to assess the response to the steroid therapy (6 scans).

In three cases initial PET scan confirmed active cardiac disease—two patients with both heart and lymph nodes involvement, one with active lesions only in the heart. In four patients, active lung and lymph node sarcoidosis was diagnosed (heart disease excluded). Four cases were negative and two revealed pneumonia. Two cases negative for heart sarcoidosis were discordant with prior MRI results.

Further decisions were made by clinical consensus, which took PET results into consideration.

**Fig. 4:**
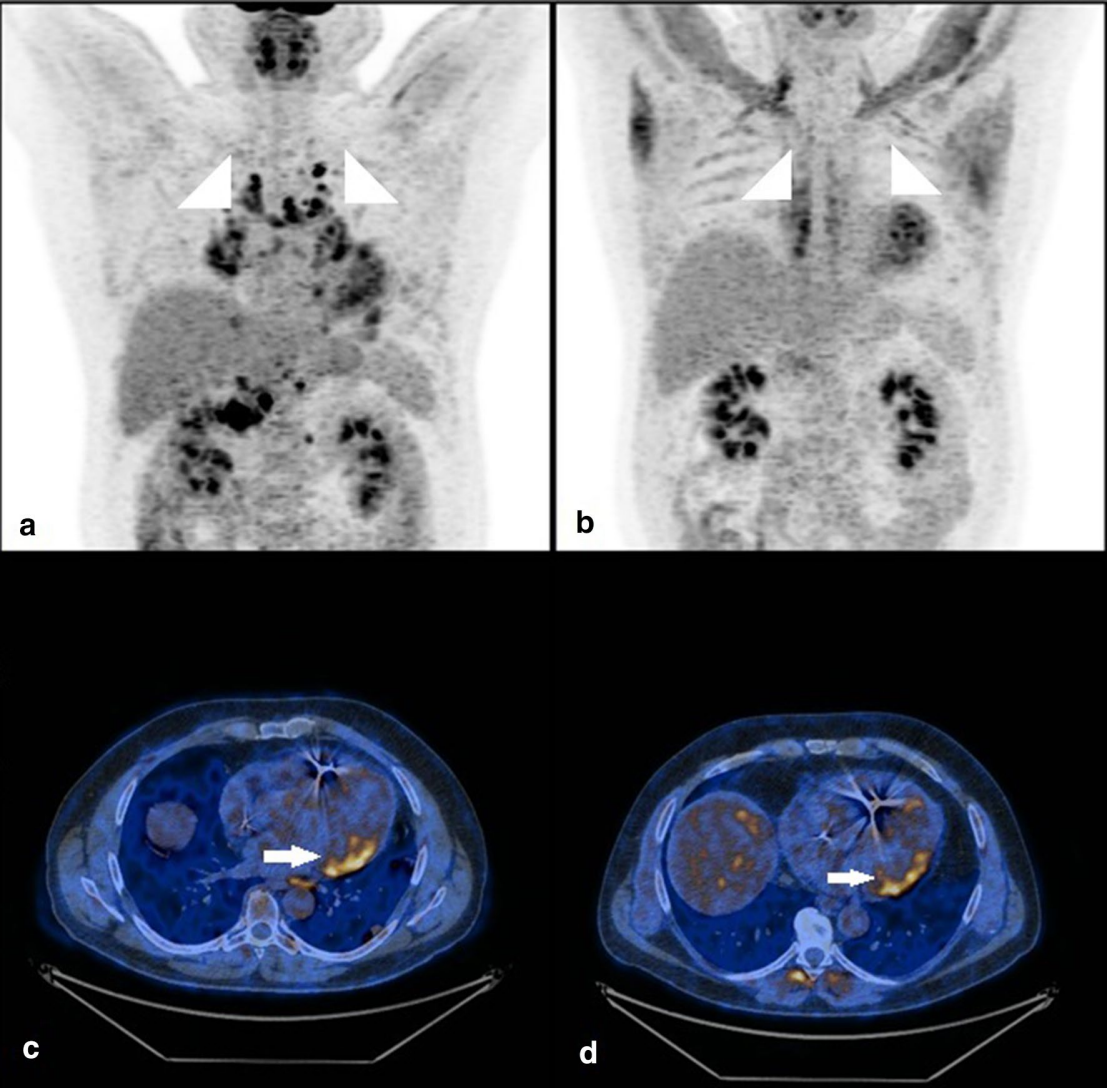
A 54-year-old patient with sarcoidosis. Primary scan (**a** maximum intensity projection (MIP) and **c** axial) revealed an increased uptake foci in the inferior lateral segments of the left ventricle (concordant with prior magnetic resonance imaging findings) and active mediastinal lymph nodes. The follow-up scan (**b**, **d**) after 4 months of steroid treatment shows good response in lymph nodes, but no significant changes in the heart

Six scans were performed to assess the treatment response. In one patient (Fig. 4) initial study confirmed active cardiac lesions (visualised in prior MRI) and lung lesions. The follow-up study after steroid therapy showed improvement in the lungs, but the disease in the heart was still active. Other patient had initially had some active lesions in the lungs and the heart. His first follow-up scan showed good response to the treatment in the lungs but was non-diagnostic for the heart assessment due to a high physiological FDG uptake. Scan was repeated study after better patient preparation and showed good response to the treatment in both heart and lungs. Three patients were referred for the treatment response assessment, but had no initial scan available. One of them had a suspicion of cardiac involvement in echocardiography and he had contradictions to MRI. The ^18^FDG PET revealed no ^18^FDG-avid lesions neither in the heart nor in the lungs which was indicative of a good response to the treatment. The second patient scan was non-diagnostic due to the high physiological ^18^FDG uptake in myocardium. The third patient had no ^18^FDG-avid foci visible which indicated a good response to the treatment.

### Delayed study

**Fig. 5:**
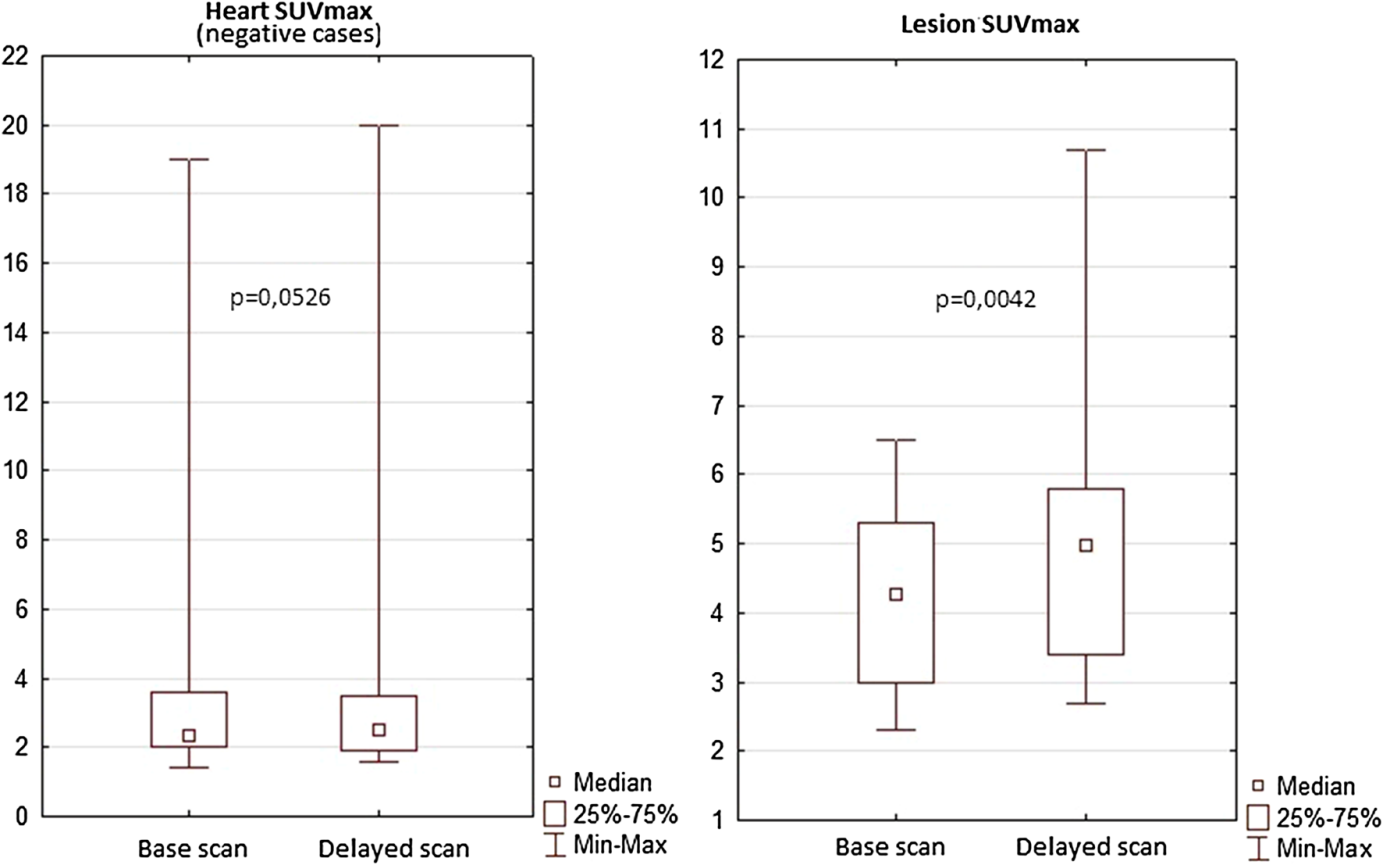
SUV comparison between the baseline scan and the delayed scan

All patients had a standard acquisition 45–90 min after radiotracer injection and another—delayed acquisition an hour after the standard scan. In the positive scans SUVmax values increase was observed in the delayed study. In the negative cases no significant differences between the standard and delayed scans were found (Fig. 5). However these differences had no impact on final conclusions, as baseline study already provided satisfactory foci delineation, with no additional lesions revealed in the delayed scans.

## Discussion

The literature on the utility of the ^18^FDG PET in the endocarditis diagnosis is scant. It is known to have a high negative predictive value up to 100% [5]. Also, an increased ^18^FDG avidity around a prosthetic valve is considered to be one of the major criteria of endocarditis in the European guidelines [4]. The diagnosis must be very careful, because it leads to the implant extraction which, for obvious reasons, may have severe complications. Our study confirms that PET is very useful in localising inflammatory foci in endocarditis. In our study it confirmed the diagnosis, found the source of inflammation (even when it was located outside of the heart) and indicated which prosthesis is infected in case of patients with multiple grafts.

In our study in 36% of patients with suspected endocarditis PET changed the initial diagnosis, recognising the source of extracardiac infection. Although our study focused on the heart diseases, results confirm that nuclear medicine has a lot to offer to the patients with fever of unknown origin. In the literature ^18^FDG PET can establish a correct diagnosis in about 50% of cases [6, 7]. In the subgroup of patients with suspected endocarditis that had been included in our analysis PET result was positive for the inflammation in nine out of fourteen patients, interestingly only four patients had the endocarditis.

Sarcoidosis is a complex disease in which granulomas can form in the heart. The steroid therapy of an active disease can prevent complications such as heart failure [3]. The challenge is to distinguish an active disease from the scarring process. In our group most patients were referred to the PET scan for that reason—they had disease in the lungs and/or lymph nodes and a suspected heart involvement. PET helped to diagnose an active heart disease also giving a valuable information to the clinicians.

In both cases of heart inflammation mentioned above the treatment response assessment is a common indication for ^18^FDG PET—morphological changes are often difficult to localise and MRI lacks specificity to distinguish an active granuloma from a scar [3]. In our group the PET scan was discordant with MRI twice in the primary diagnosis. As a follow-up study PET was a valuable tool to monitor the treatment response. We observed that the dynamics of the inflammatory lesions in the lungs, lymph nodes and heart may vary and that the remission in one organ is not necessarily a sign of a therapeutic success.

What is challenging in the cardiac nuclear imaging is the patient preparation. For a diagnostic PET scan patients need to be fasting to switch cardiac metabolism from glucose to fatty acids. We observed two cases of a non-diagnostic scan due to the high physiologic metabolism of ^18^FDG in the myocardium. One of these patients did not have a scan repeated yet, another one had a good quality PET images after an appropriate preparation. Another limitation to the ^18^FDG PET imaging is a 3 to 6 months period after surgery when we cannot distinguish an inflammatory activity from reactive (postoperative) changes.

The examination protocol usually requires the images to be acquired 60–90 min after the radiotracer injection. Some departments (including ours) perform an additional delayed scan 1 h after the standard one. In our group, however, the delayed scan had no significant impact on the final diagnosis. Yet, in the delayed scans the increased SUV values and a better delineation of ^18^FDG-avid foci could be observed.

## Conclusions

The ^18^FDG PET is a valuable method for the cardiac inflammation assessment. It localises the active inflammation well. However, a good patient preparation is essential for suppressing the physiological ^18^FDG uptake in the myocardium. An important advantage of PET is that it is a whole-body scan that enables to localise extracardiac lesions, which in turn may lead to the notable change of the initial diagnosis. It is also a useful method of assessing the response to the treatment. The delayed cardiac scans showed no additional value to the standard scans in the investigated group.
